# Association between glycemic control and chronic kidney disease development in older patients with type 2 diabetes: A retrospective cohort study

**DOI:** 10.1371/journal.pone.0353388

**Published:** 2026-07-16

**Authors:** Mar Riera-Pagespetit, Gorka Gómez-Terrazas, Doris Xiomara Monroy-Parada, Cristian Tebé, Carlos Pérez-López, Silvia Miró Cañís, María José Castro-Castro, Anna Cortés-Bosch de Basea, Alejandro Rodríguez-Molinero

**Affiliations:** 1 Geriatrics Department, Consorci Sanitari de l’Alt Penedès i Garraf, Vilafranca del Penedès, Spain; 2 Emergency Department, Hospital Universitario de Cruces, Barakaldo, Spain; 3 SAP Baix Llobregat Centre, Direcció d’Atenció Primària Metropolitana Sud, Gerència Territorial Metropolitana Sud, Institut Català de la Salut, Cornellà de Llobregat, Barcelona, Spain; 4 Biostatistics Support and Research Unit, Germans Trias I Pujol Research Institute and Hospital (IGTP), Campus Can, Badalona, Spain; 5 Research Department, Consorci Sanitari de l’Alt Penedès i Garraf, Vilafranca del Penedès, Spain; 6 Clinical Analysis Laboratory CLILAB Diagnòstics, Vilafranca del Penedès, Spain; 7 Laboratori Clínic, Hospital Universitari de Bellvitge, L’Hospitalet de Llobregat, Spain; The Chinese University of Hong Kong, HONG KONG

## Abstract

**Background:**

In older patients with type 2 diabetes (T2D), physicians often de-emphasize strict glycemic control to avoid severe hypoglycemia. However, the incidence of renal impairment in diabetic octogenarians is underexplored, and the impact of glycemic control on kidney function in this age group remains unclear. This study assessed its effect on CKD development.

**Methods:**

Retrospective, multicenter cohort study including patients (>80 years) with T2D between 2012 and 2016, at least one annual glycated hemoglobin (HbA1c) measurement, an estimated glomerular filtration rate (GFR) ≥60 mL/min/1.73m^2^, and ≥one annual GFR estimation during follow-up. Patients were classified according to glycemic control (poor and good); good control was set at HbA1c < 7.5%. Five-year follow-up data were collected from medical records.

**Results:**

The study included 1062 patients, 435 (40.96%) in the poor glycemic control group and 627 (59.04%) in the good control group. CKD incidence was significantly higher among individuals with poor control (61.61%) compared to those with good control (52.47%) (p = 0.003). Logistic regression analyses showed that poor control is independently associated with higher odds of CKD onset (OR: 0.87 good vs. poor control, p = 0.010) and accelerates its progression (time-to-CKD HR: 0.78 good vs. poor control, p = 0.004).

**Conclusions:**

Poor glycemic control is independently associated with CKD development and a shorter time to CKD onset, supporting its potential relevance for kidney-related outcomes in older patients with T2D. These findings highlight the need to consider glycemic control within a broader, individualized clinical approach in this population.

## Introduction

Type 2 diabetes (T2D) is a growing chronic condition, especially in developed countries, and its prevalence increases with age [1]. The latest dataset from the Global Burden of Disease (GBD) review in 2017 indicates that T2D affected approximately 462 million individuals, 6.28% of the global population, and was directly linked to over one million deaths annually, categorizing it as the ninth primary cause of mortality worldwide [2]. Type 2 diabetes results in high glucose concentration in plasma/serum (hyperglycemia) [3], leading to various complications such as retinopathy, neuropathy, nephropathy, and macrovascular issues [4].

Factors associated with T2D include reduced insulin secretion, insulin resistance, muscle loss (sarcopenia), and lack of physical activity, particularly among older patients [3]. In older adults, T2D increases the likelihood of physical disability and is an independent risk factor for falls and hip fractures [5]. Geriatric patients with T2D have higher rates of premature death and an increased incidence of health conditions, such as hypertension, heart disease, cerebrovascular disease, and stroke compared to those without T2D. In addition, they are more susceptible to common geriatric syndromes, like polypharmacy, depression, cognitive decline, urinary incontinence, falls resulting in injuries, and persistent pain [4].

Diabetes is the most common risk factor for developing chronic kidney disease (CKD) [6], defined as a low estimated glomerular filtration rate (<60 mL/min/1.73 m^2^) for at least three months or evidence of kidney damage [7]. Patients with CKD are at risk of cardiovascular disease and death, and upon progression to end stage, treatment options are limited [8]. The estimated global prevalence of CKD ranges from 8% to 16% [9]. In Spain, it is 9.2% among adults, increasing to 20.6% in individuals over 64 years, likely due to aging and cardiovascular risk factors [10]. Compared to non-diabetic patients, individuals with diabetes have a notably elevated risk of developing end-stage renal disease, making it the most critical risk factor for the development of CKD in developed countries [11]. Furthermore, the concurrent presence of T2D and CKD significantly amplifies the likelihood of cardiovascular disease [6].

Despite the increased morbimortality risks of older patients with T2D [12–14], physicians often adopt a more lenient approach to glucose concentration in plasma/serum management in this demographic group, assuming that severe diabetes complications might not develop quickly enough to affect their projected lifespan. Moreover, physicians de-emphasize strict glucose control in the older patients primarily to avoid the severe consequences of hypoglycemia. Hypoglycemic episodes, prevalent among the older patients, can lead to falls and physical and cognitive impairments, increasing their risk of disability and death [15]. However, with life expectancies on the rise, especially in high-income countries [16], it is essential to reconsider this approach. In this regard, no studies have specifically analyzed the incidence of kidney impairment in diabetic patients aged 80 years and older, and the impact of glycemic control on CKD development in this age group with T2D remains unknown.

This retrospective study aimed to investigate whether the incidence of CKD in older patients (>80 years) with T2D is related to diabetes control, measured by the glycated hemoglobin fraction in blood (HbA1c). The secondary objectives of this study were to explore the association between the rate of decline in renal function among older patients with T2D and diabetes control, measured by HbA1c fraction.

## Materials and methods

### Study design and population

This was a retrospective, multicenter cohort study using data from the electronic medical records of patients attending the primary care services of the Garraf region in Catalonia (Garraf, Alt Penedès y Baix Llobregat; South region of Barcelona, Catalonia, Spain). Our study included patients aged 80 years or older who were diagnosed with type 2 diabetes (T2D) one year before inclusion and were alive during the study period. These patients were required to have at least one annual measurement of HbA1c fraction, an estimated glomerular filtration rate (GFR) equal to or greater than 60 mL/min/1.73m^2^ at baseline, and at least one annual GFR estimation during the follow-up period. All patients meeting these inclusion criteria from 2012 to 2016 were consecutively included in the study, starting from 2016 and backward until reaching the estimated sample size. Retrospective follow-up data spanning five years were collected.

The need for informed consent was waived by the Institut Universitari d’Investigació en Atenció Primària Jordi Gol i Gurina (IDIAPJGol; reference number: 21/155-P), which approved the study. This research adhered to the principles outlined in the Helsinki Declaration and complied with the EU General Data Protection Regulation (GDPR). As per GDPR guidelines, all personal data were appropriately anonymized and kept separate from the research results.

### Data source

In this retrospective cohort study, all data were extracted from preexisting sources of information generated for clinical care. The databases used included the computerized database of clinical analyses of the Consorci del Laboratori Intercomarcal de L’Alt Penedés L’Anoia i El Garraf (CLI), the computerized database of clinical analyses of the Bellvitge University Hospital (hematology and biochemistry areas), and the eCAP, which is a computerized clinical history software and database used by health professionals of the Institut Català de la Salut (ICS) during patient visits.

The data was extracted by a team independent of the research team, and the researchers did not have access to personally identifiable information. The researchers first accessed the data on October 4, 2021.

### Objectives and variables

To calculate the incidence of CKD (primary objective), CKD was defined as at least two analytical determinations with estimated glomerular filtration rate (GFR) < 60 mL/min/1.73m2 during follow-up (using CKD-EPI equation). Although at least one HbA1c measurement per year was required for inclusion, glycemic control was defined using all available HbA1c measurements throughout the follow-up period, allowing for a longitudinal assessment of glycemic exposure. Participants were categorized based on the level of control of their T2D, determined by their HbA1c fraction (%). Two cohorts were established: (1) the good glycemic control group comprised patients who predominantly exhibited HbA1c fraction of 7.5% or lower throughout the follow-up period. Moreover, they did not have two consecutive HbA1c determinations surpassing this threshold. (Consecutive determinations were defined as those separated by a minimum of three months, while two consecutive determinations occurring within a shorter interval were considered a single determination); (2) the poor glycemic control group included patients who primarily showed HbA1c fraction above 7.5%, as well as individuals who had at least two consecutive HbA1c determinations exceeding the 7.5% limit. The HbA1c cut-off of 7.5% was based on guideline recommendations for older adults [17].

Other variables extracted from clinical records included sex, age, year of inclusion, previous comorbidities (dyslipidemia, hypertension, obesity, smoking, and alcohol consumption), and previous pharmacological treatments (statins, angiotensin-converting enzyme [ACE] inhibitors, oral antidiabetics, non-steroidal anti-inflammatory drugs [NSAIDs], insulin, and angiotensin receptor blockers [ARBs]).

### Sample size calculation

A preliminary analysis of the available data indicated that, within the study’s specified period and geographic scope, data from 2257 older individuals with diabetes aged 80 years or older were available. Assuming a prevalence rate of renal failure at 40%, 1354 older individuals without prior renal failure would be included in the study.

The anticipated event rate, specifically renal failure at 5 years, was projected to be 27% in the group with poor glycemic control and 18% in the group with good glycemic control. By assuming a hazard ratio (HR) of 1.5 and a type I error rate of 5%, the statistical power to reject the null hypothesis of equal survival curves exceeds 80% with a sample size of 1200 patients, regardless of the patients’ distribution between the groups being at 2:1 or 3:1 ratio.

### Statistical analysis

Categorical variables were described as frequencies and percentages, and continuous variables were described as the mean and standard deviation (SD).

To determine whether glycemic control is associated with CKD incidence, we constructed a multivariate logistic regression model. The selection of variables included in the multivariate models was based on their statistical significance in bivariate analyses and clinical relevance. The log-rank test was used to estimate CKD as a function of time (years) and a Cox-regression analysis was used to determine whether glycemic control is associated with time to CKD onset. Odds Ratio (ORs) and HRs were calculated along with their 95% confidence interval (CI) and *p*-values. The threshold for statistical significance in all analyses was set at a two-sided alpha (α) < 0.05 (*p* < 0.05). All statistical analyses were performed using the R 4.1.0 software.

## Results

### Demographic and clinical characteristics of study patients

A total of 1062 patients were included in this study, with 435 (40.96%) patients in the poor glycemic control group and 627 (59.04%) in the good glycemic control group. The complete demographic and clinical characteristics of the two patient groups are summarized in Tables 1 and 2, respectively.

**Table 1:** Patients’ demographic characteristics according to the study group.

	Poor glycemic control (n = 435)	Good glycemic control (n = 637)
**Gender, *n (%)***		
Female	261 (60.00)	390 (62.20)
Male	174 (40.00)	237 (37.80)
**Age at inclusion (years), *mean (SD)***	88.89 (2.88)	88.90 (2.99)
**Inclusion year, *n (%)***		
2012	97 (22.30)	27 (4.31)
2013	42 (9.66)	91 (14.51)
2014	73 (16.78)	144 (22.97)
2015	54 (12.41)	83 (13.24)
2016	169 (38.85)	282 (44.98)
**Years from diagnosis to start of follow-up, *median (IQR)***	10.50 (7.95, 13.11)	9.36 (5.60, 12.43)

IQR, interquartile range; SD, standard deviation.

**Table 2:** Clinical and treatment characteristics of study patients, *n (%).*

	Poor glycemic control (n = 435)	Good glycemic control (n = 637)
**Clinical characteristics**		
Dyslipidemia	229 (52.64)	367 (58.53)
Hypertension	365 (83.91)	522 (83.25)
Obesity	131 (30.11)	228 (36.36)
Smoking	49 (11.26)	60 (9.57)
Alcohol consumption	2 (0.46)	3 (0.48)
**Previous treatments**		
Statins	45 (10.34)	81 (12.92)
ACE inhibitors	21 (4.83)	51 (8.13)
Oral antidiabetics	89 (20.46)	172 (27.43)
NSAIDs	8 (1.84)	11 (1.75)
Previous insulin	36 (8.28)	9 (1.44)
ARBs	10 (2.30)	20 (3.19)

ACE, angiotensin-converting enzyme; ARBs, angiotensin receptor blockers; NSAIDs, non-steroidal anti-inflammatory drugs.

### Incidence of CKD and glycemic control

In the raw analysis, among participants with poor glycemic control (n = 435), 268 CKD events were documented, resulting in a 61.61% (95% CI: 56.86–66.20) incidence during the 5-year follow-up. Conversely, in the group with good glycemic control (n = 627), 329 CKD events were documented, resulting in a 52.47% (95% CI: 48.48–56.44) incidence. The incidence rates of CKD were significantly different between the two groups (*p* = 0.003), indicating that individuals with poor glycemic control showed a significantly higher incidence of CKD than those with good glycemic control.

### Poor glycemic control as a risk factor for CKD

Table 3 presents the results of the logistic regression model analyzing the adjusted association between glycemic control and CKD incidence. After adjustment for age, gender, previous dyslipidemia, previous use of statins, ACE inhibitors, oral antidiabetic medications, insulin, and ARBs, the protective effect of good glycemic control remained statistically significant (OR=0.87, 95% CI: 0.78–0.97, p = 0.010).

**Table 3:** Logistic regression analysis for developing chronic kidney disease.

Predictors	Odds ratio	95% CI	*p*-value
**Strict glycemic control**	0.87	0.78–0.97	**0.010**
**Adusting covariates**			
Age	1.02	1.01–1.04	**0.006**
Gender (male)	1.06	0.95–1.18	0.274
Previous hypertension	1.25	1.06–1.48	**0.009**
Previous obesity	1.07	0.96–1.19	0.242
Previous dyslipidemia	0.99	0.89–1.09	0.779
Years from diagnosis to start of follow-up	1.01	1.00–1.01	**0.023**
Previous statins	0.88	0.73–1.06	0.163
Previous ACE	0.97	0.79–1.20	0.797
Previous oral antidiabetics	0.90	0.79–1.03	0.136
Previous insulin	0.84	0.64–1.11	0.216
Previous ARBs	0.90	0.65–1.24	0.519

ACE, angiotensin-converting enzyme; ARBs, angiotensin receptor blockers; CI, confidence interval; NSAIDs, non-steroidal anti-inflammatory drugs.

R^2^ Nagelkerke = 0.043.

Poor glycemic control was used as the reference (OR: 1). Bold figures indicate statistical significance (*p* < 0.05).

### Survival analysis of CKD incidence

**Fig 1:**
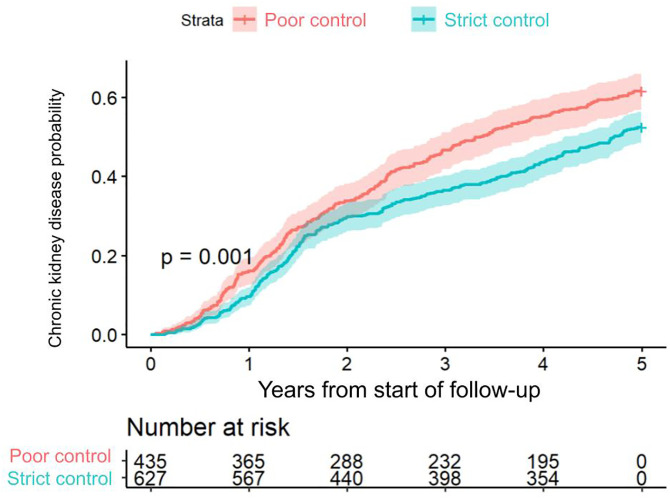
Kaplan-Meier analysis of the probability of chronic kidney disease as a function of the years since start of follow-up. The p-value was calculated using the log-rank test.

The total number of observed patient-years was 1382.3 in the group of patients with poor glycemic control and 2222.8 in the group of patients with good glycemic control. Survival analysis of the time to CKD from start of follow-up (years) showed that patients in poor glycemic control had a significantly higher incidence rate of CKD (193.88 per 1000 patient-year, 95% CI: 171.36, 218.15) compared to those with good glycemic control (148.01 per 1000 patient-year, 95% CI: 132.45, 164.66) (p = 0.001) (Fig 1).

Table 4 presents the results of the Cox regression model analyzing the adjusted association between glycemic control and time to CKD. After adjusting for the previous described factors, the protective effect of good glycemic control remained statistically significant (HR = 0.78, 95% CI: 0.66–0.93, p = 0.004)

**Table 4:** Adjusted Cox-regression analysis of time to chronic kidney disease from start of follow-up as a function of study group.

	Hazard ratio	95% CI	*p*-value
**Strict glycemic control**	0.78	0.66–0.93	**0.004**
**Adjusting covariates**			
Age	1.04	1.01–1.07	**0.008**
Gender (male)	1.04	0.88–1.24	0.613
Previous hypertension	1.40	1.11–1.78	**0.005**
Previous obesity	1.11	0.94–1.32	0.221
Previous dyslipidemia	0.98	0.83–1.15	0.787
Years from diagnosis to start of follow-up	1.08	1.00–1.17	**0.045**
Previous statins	0.80	0.61–1.05	0.109
Previous ACE	0.88	0.63–1.22	0.434
Previous oral antidiabetics	0.81	0.66–1.00	0.052
Previous insulin	0.74	0.48–1.13	0.164
Previous ARBs	0.86	0.52–1.40	0.537

ACE, angiotensin-converting enzyme; ARBs, angiotensin receptor blockers; CI, confidence interval.

R^2^ Nagelkerke = 0.036.

Poor glycemic control was used as the reference (HR: 1). Bold figures indicate statistical significance (*p* < 0.05).

## Discussion

To our knowledge, this is the first study examining the incidence of CKD based on glycemic control in a population of T2D patients aged over 80 years. Our retrospective cohort analysis revealed a notable increase in the incidence of CKD among those with poor glycemic control compared to those with good glycemic control. In the adjusted logistic regression analysis, poor glycemic control was identified as an independent factor associated with increased risk of developing CKD and shorter time to CKD Previously identified factors, including age, previous hypertension, and increased years from diagnosis to start of follow-up also showed significant associations. Interestingly, some comorbidities and treatments were slightly more prevalent in the group with better glycemic control, possibly reflecting differences in healthcare utilization and closer clinical follow-up in these patients, although this interpretation remains speculative. The time to CKD was significantly shorter in patients with poor glycemic control compared to those with good glycemic control.

In this study, we established the threshold of HbA1c at 7.5% to differentiate between poor and good glycemic control. However, in the context of the older population, there is currently no consensus on a standard threshold for defining normal HbA1c fraction. The existing guidelines, including those provided by the American Geriatrics Society (AGS) [18], the American Diabetes Association (ADA) [17], the International Diabetes Federation (IDF) [19], and the European Diabetes Working Party [20], advocate for an individualized approach to determine the appropriate HbA1c target. This approach considers the patient’s overall health, life expectancy, specific risks of hypoglycemia, and ability to adhere to treatment regimens. The recommended target range for HbA1c is usually between 7% and 8.5%, with lower targets recommended for younger and healthier patients and higher targets for older patients with multiple comorbidities. The ACCORD (Action to Control Cardiovascular Risk in Diabetes) study aimed to assess whether intensive glycemic control (HbA1c < 6%) compared to standard control (HbA1c between 7%−7.9%) would lead to favorable cardiovascular outcomes in individuals between 40 and 79 years with T2D and high vascular risk [21]. This study established that an HbA1c target of 7.0%−7.9% (with a mean of 7.5%) might be safer for patients with long-standing T2D and a high risk of cardiovascular disease. Considering all available information and the characteristics of our study population (> 80 years), we established a threshold of 7.5% to classify patients according to glycemic control (good vs. poor).

However, the use of a single threshold to categorize glycemic control may oversimplify what is inherently a continuous variable and could lead to potential misclassification, particularly for individuals with HbA1c values close to the selected cut-off. Alternative approaches, such as using multiple categories or analyzing HbA1c as a continuous variable, might provide a more nuanced understanding of the relationship between glycemic control and renal outcomes. Nevertheless, given the characteristics of our study population and the available sample size, a binary classification was considered a pragmatic approach to ensure statistical robustness and facilitate clinical interpretability.

This study showed an increased incidence and shorter time of developing CKD in individuals with poor glycemic control. Although this research is the first that focuses on older patients (>80 years), other studies have analyzed the relationship between the development of CKD and glycemic control. Specifically, our results align with current scientific evidence, consistently demonstrating a strong association between CKD and poor glycemic control in adult patients [22]. Research consistently indicates that uncontrolled or poorly managed diabetes elevates CKD risk due to prolonged high blood glucose levels. This connection is supported by epidemiological evidence linking poor glycemic control with microvascular complications in type 2 diabetes, which are considered a key factor in CKD onset [22–25]. Irrespective of the mechanisms linking both conditions, the findings of this study highlight a risk of CKD in older patients with poor glycemic control. While the risk of hypoglycemia associated with glycemic control interventions in these individuals should be kept in mind [15], it is crucial to consider the risks of inadequate glycemic control. Poor glycemic control is associated with increased likelihood of developing CKD, with potentially severe consequences, such as the need for renal replacement therapies, which significantly impact both health-related quality of life and mortality rates [26]. Our findings further reinforce this perception, highlighting the negative consequences of inappropriate or non-strict T2D management.

The interpretation of this study’s results should consider certain limitations, primarily stemming from the retrospective nature of the research [27]. However, key aspects of the study strengthen the reliability of the results. Both the main exposure (HbA1c) and outcome (glomerular filtration rate) were based on routinely collected analytical measurements, which are highly reproducible and less prone to observer-related bias [28]. In addition, the data analysis investigators were blinded to the glycemic control group.

One limitation of this study relates to the potential influence of residual confounding, particularly due to cardiovascular conditions that were not explicitly captured in our dataset. Although we adjusted our analyses for several related variables, including hypertension, dyslipidemia, and pharmacological treatments (such as angiotensin-converting enzyme inhibitors and angiotensin receptor blockers), we cannot exclude the possibility of residual confounding due to the lack of direct information on cardiovascular disease. Therefore, our findings should be interpreted with caution, and future studies incorporating more detailed cardiovascular data are needed to better disentangle these relationships.

In addition, we did not have information on proteinuria, an important marker of kidney damage and a well-established predictor of CKD progression. Although its inclusion could have provided additional clinical context, the role of proteinuria in this setting is complex, as it is likely to represent, at least in part, a downstream manifestation of chronic hyperglycemia and may lie along the causal pathway linking glycemic control and kidney function decline. Therefore, adjusting for proteinuria could have led to an underestimation of the association of interest.

An other potential limitation of the study lies in the exclusion of patients who died during the follow-up period. This exclusion introduces a potential bias, influencing the overall incidence of CKD observed among patients who were alive during the five-year follow-up. To address this concern, we additionally analyzed glycemic control among patients with exitus during the follow-up period, ruling out a significant association between mortality events and glycemic control (S1 Table). Overall, these limitations should be considered when interpreting the findings.

## Conclusions

In conclusion, this study is the first to comprehensively examine the impact of glycemic control on the incidence of CKD in an older population aged ≥80 years. The results of this retrospective cohort study suggest that poor glycemic control is associated with a higher risk and earlier onset of CKD, highlighting that glycemic management remains clinically relevant even in very old adults.

However, given the complexity of T2D management in this population, these findings should not be interpreted as supporting a universal strategy of tighter glycemic control. In individuals aged ≥80 years, glycemic targets should remain individualized, taking into account frailty, functional status, comorbidities, and the risk of hypoglycemia. Therefore, the potential benefits of improved glycemic control on renal outcomes must be carefully balanced against the risks associated with overtreatment.

Future studies assessing other T2D-related outcomes and incorporating broader geriatric parameters are essential to confirm these findings and to help inform more tailored approaches for optimal T2D management in this age group.

## Supporting information

S1 Table Analysis of mortality incidence and its association with glycemic control. (DOCX)
