# Supplementary material for: Association between glycemic control and chronic kidney disease development in older patients with type 2 diabetes: A retrospective cohort study
Source: PLoS One. 2026 Jul 16;21(7):e0353388. doi: 10.1371/journal.pone.0353388 (PMC13374871; doi:10.1371/journal.pone.0353388)
Supplement: S1 Table — (DOCX) [file pone.0353388.s001.docx]

**Supplementary Table 1.** Analysis of mortality incidence and its association with glycemic control.

|  | **Good glycemic control** | **Poor glycemic control** | ***p*-value*** |
| --- | --- | --- | --- |
| **Exitus, n** | 168 (38.3%) | 94 (38.7%) | 0.98 |
| **Alive, n** | 271 (61.7) | 149 (61.3%) |  |

* The chi-square statistic with Yates correction. The inclusion and exclusion criteria for this analysis were consistent with those of the overall study, with the exception of the requirement that participants must have been alive during the entire study period.
