# Supplementary material for: Association between glycemic control and chronic kidney disease development in older patients with type 2 diabetes: A retrospective cohort study
Source: PLoS One. 2026 Jul 16;21(7):e0353388. doi: 10.1371/journal.pone.0353388 (PMC13374871; doi:10.1371/journal.pone.0353388)
Supplement: Peer review history

# Peer review: Decision Letter 0

**DOI:** 10.1371/journal.pone.0353388.r001

**Authors:** Natural Hoi Sing Chu

12 Mar 2026

PONE-D-25-64900

Association Between Glycemic Control and Renal Function in Older Patients with Type 2 Diabetes: A Retrospective Cohort Study

PLOS One

Dear Dr. Rodríguez-Molinero,

Thank you for submitting your manuscript to PLOS ONE. After careful consideration, we feel that it has merit but does not fully meet PLOS ONE’s publication criteria as it currently stands. Therefore, we invite you to submit a revised version of the manuscript that addresses the points raised during the review process.

Please submit your revised manuscript by Apr 26 2026 11:59PM. If you will need more time than this to complete your revisions, please reply to this message or contact the journal office at plosone@plos.org. When you're ready to submit your revision, log on to https://www.editorialmanager.com/pone/ and select the 'Submissions Needing Revision' folder to locate your manuscript file.

Please include the following items when submitting your revised manuscript:

- A letter that responds to each point raised by the academic editor and reviewer(s). You should upload this letter as a separate file labeled 'Response to Reviewers'.
- A marked-up copy of your manuscript that highlights changes made to the original version. You should upload this as a separate file labeled 'Revised Manuscript with Track Changes'.
- An unmarked version of your revised paper without tracked changes. You should upload this as a separate file labeled 'Manuscript'.

-->If you would like to make changes to your financial disclosure, please include your updated statement in your cover letter. Guidelines for resubmitting your figure files are available below the reviewer comments at the end of this letter.

If applicable, we recommend that you deposit your laboratory protocols in protocols.io to enhance the reproducibility of your results. Protocols.io assigns your protocol its own identifier (DOI) so that it can be cited independently in the future. For instructions see: https://journals.plos.org/plosone/s/submission-guidelines#loc-laboratory-protocols. Additionally, PLOS ONE offers an option for publishing peer-reviewed Lab Protocol articles, which describe protocols hosted on protocols.io. Read more information on sharing protocols at https://plos.org/protocols?utm_medium=editorial-email&utm_source=authorletters&utm_campaign=protocols.

We look forward to receiving your revised manuscript.

Kind regards,

Natural Hoi Sing Chu, Ph.D

Academic Editor

PLOS One

Journal Requirements:

When submitting your revision, we need you to address these additional requirements.

1. Please ensure that your manuscript meets PLOS ONE's style requirements, including those for file naming. The PLOS ONE style templates can be found at

https://journals.plos.org/plosone/s/file?id=wjVg/PLOSOne_formatting_sample_main_body.pdf and https://journals.plos.org/plosone/s/file?id=ba62/PLOSOne_formatting_sample_title_authors_affiliations.pdf

2. We note that you have indicated that there are restrictions to data sharing for this study. For studies involving human research participant data or other sensitive data, we encourage authors to share de-identified or anonymized data. However, when data cannot be publicly shared for ethical reasons, we allow authors to make their data sets available upon request. For information on unacceptable data access restrictions, please see http://journals.plos.org/plosone/s/data-availability#loc-unacceptable-data-access-restrictions.

Before we proceed with your manuscript, please address the following prompts:

a) If there are ethical or legal restrictions on sharing a de-identified data set, please explain them in detail (e.g., data contain potentially identifying or sensitive patient information, data are owned by a third-party organization, etc.) and who has imposed them (e.g., a Research Ethics Committee or Institutional Review Board, etc.). Please also provide contact information for a data access committee, ethics committee, or other institutional body to which data requests may be sent.

b) If there are no restrictions, please upload the minimal anonymized data set necessary to replicate your study findings to a stable, public repository and provide us with the relevant URLs, DOIs, or accession numbers. Please see http://www.bmj.com/content/340/bmj.c181.long for guidelines on how to de-identify and prepare clinical data for publication. For a list of recommended repositories, please see https://journals.plos.org/plosone/s/recommended-repositories. You also have the option of uploading the data as Supporting Information files, but we would recommend depositing data directly to a data repository if possible.

Please update your Data Availability statement in the submission form accordingly.

3. In the online submission form, you indicated that the datasets generated and/or analyzed during the current study are available from the corresponding author on reasonable request.

All PLOS journals now require all data underlying the findings described in their manuscript to be freely available to other researchers, either 1. In a public repository, 2. Within the manuscript itself, or 3. Uploaded as supplementary information.

This policy applies to all data except where public deposition would breach compliance with the protocol approved by your research ethics board. If your data cannot be made publicly available for ethical or legal reasons (e.g., public availability would compromise patient privacy), please explain your reasons on resubmission and your exemption request will be escalated for approval.

4. We note that there is identifying data in the Supporting Information file <renamed_0029b.pdf>. Due to the inclusion of these potentially identifying data, we have removed this file from your file inventory. Prior to sharing human research participant data, authors should consult with an ethics committee to ensure data are shared in accordance with participant consent and all applicable local laws.

Data sharing should never compromise participant privacy. It is therefore not appropriate to publicly share personally identifiable data on human research participants. The following are examples of data that should not be shared:

-Name, initials, physical address

-Ages more specific than whole numbers

-Internet protocol (IP) address

-Specific dates (birth dates, death dates, examination dates, etc.)

-Contact information such as phone number or email address

-Location data

-ID numbers that seem specific (long numbers, include initials, titled “Hospital ID”) rather than random (small numbers in numerical order)

Data that are not directly identifying may also be inappropriate to share, as in combination they can become identifying. For example, data collected from a small group of participants, vulnerable populations, or private groups should not be shared if they involve indirect identifiers (such as sex, ethnicity, location, etc.) that may risk the identification of study participants.

Additional guidance on preparing raw data for publication can be found in our Data Policy (https://journals.plos.org/plosone/s/data-availability#loc-human-research-participant-data-and-other-sensitive-data) and in the following article: http://www.bmj.com/content/340/bmj.c181.long.

Please remove or anonymize all personal information (<specific identifying information in file to be removed>), ensure that the data shared are in accordance with participant consent, and re-upload a fully anonymized data set. Please note that spreadsheet columns with personal information must be removed and not hidden as all hidden columns will appear in the published file.

5. Please include captions for your Supporting Information files at the end of your manuscript, and update any in-text citations to match accordingly. Please see our Supporting Information guidelines for more information: http://journals.plos.org/plosone/s/supporting-information.

6. If the reviewer comments include a recommendation to cite specific previously published works, please review and evaluate these publications to determine whether they are relevant and should be cited. There is no requirement to cite these works unless the editor has indicated otherwise.

7. Please review your reference list to ensure that it is complete and correct. If you have cited papers that have been retracted, please include the rationale for doing so in the manuscript text, or remove these references and replace them with relevant current references. Any changes to the reference list should be mentioned in the rebuttal letter that accompanies your revised manuscript. If you need to cite a retracted article, indicate the article’s retracted status in the References list and also include a citation and full reference for the retraction notice.

[Note: HTML markup is below. Please do not edit.]

Reviewers' comments:

Reviewer's Responses to Questions

**Comments to the Author**

1. Is the manuscript technically sound, and do the data support the conclusions?

The manuscript must describe a technically sound piece of scientific research with data that supports the conclusions. Experiments must have been conducted rigorously, with appropriate controls, replication, and sample sizes. The conclusions must be drawn appropriately based on the data presented.

Reviewer #1: Yes

Reviewer #2: Partly

**********

2. Has the statistical analysis been performed appropriately and rigorously?

Reviewer #1: Yes

Reviewer #2: Yes

**********

3. Have the authors made all data underlying the findings in their manuscript fully available?

The PLOS Data policy requires authors to make all data underlying the findings described in their manuscript fully available without restriction, with rare exception (please refer to the Data Availability Statement in the manuscript PDF file). The data should be provided as part of the manuscript or its supporting information, or deposited to a public repository. For example, in addition to summary statistics, the data points behind means, medians and variance measures should be available. If there are restrictions on publicly sharing data—e.g. participant privacy or use of data from a third party—those must be specified.

Reviewer #1: No

Reviewer #2: No

**********

4. Is the manuscript presented in an intelligible fashion and written in standard English?

PLOS ONE does not copyedit accepted manuscripts, so the language in submitted articles must be clear, correct, and unambiguous. Any typographical or grammatical errors should be corrected at revision, so please note any specific errors here.

Reviewer #1: Yes

Reviewer #2: Yes

**********

5. Review Comments to the Author

Please use the space provided to explain your answers to the questions above. You may also include additional comments for the author, including concerns about dual publication, research ethics, or publication ethics. (Please upload your review as an attachment if it exceeds 20,000 characters)

Reviewer #1: The question is whether only one annual HbA1c measurement can speak about glycemic control in these patients?

Immediately upon reading the instructions, the fear of possible biases (the influence of other factors on glycemia and kidney function, especially in this fragile population) arises. Searching the literature, I find that similar topics have already been covered. It is clear that generally poor glucoregulation implies a less functioning kidney. But in the elderly, it can be due to a number of other reasons besides the glycemic profile.

Keywords: Somehow "diabetes control" and "glycemic control" sound the same, try to be more imaginative.

Maybe instead of "renal function" in the title, write "development of renal dysfunction or failure or similar"? Just a suggestion.

The binary division of glycemic control: black/white is also delicate. So, someone with an HbA1c of 7.4 has good control, while someone with 7.5 has bad control. It would be ideal to divide HbA1c differences of, for example, 0.5 into groups or classes in some other way to avoid such exclusivity, so we would get more credible results.

Or as ACCORD maybe take below 7 and above 8 and let's see for those subgroups. Again, just as a suggestion.

Is it by chance that the Abstract, Methods section, is written in a different font in the manuscript?

Please avoid writing abbreviations at the beginning of sentences.

Nicely written text but full of possible biases. We cannot draw such conclusions based on the analyzed data and without excluding potential other factors.

It is certainly interesting that there were slightly more smokers and comorbidities in the group with better glucoregulation. Perhaps due to better awareness of the disease? And more ACEi and NSAID users, and better glucoregulation and eGFR. I like that we have that data.

P16 L 286: "o" is probably missing an "r" (or).

Finally, when I read everything, I congratulate you because this work has value.

Reviewer #2: Overall a useful question to try to answer.

I don't understand why you chose the cutoff HbA1c 7.5% to differentiate between poor 251 and good glycemic control.

It seems like this was selected maybe based to get positive results? Most guidelines suggest <7% if risk of hypos and in T2DM.

Other issues is the strongest confounder in octogenerians will be IHD / heart failure. There is no data on this.

Another big concern is the absence or acknowledgement of any proteinuria data. This is known to have a strong impact on CKD progression. I understand may be unavailable due to historical data but you at least need to acknowledge this.

I am also concerned that the good control ACEi / ARB use was 11.32% vs 6.83% in poor control. This is a difference of 4.5% in ACEi /ARB use. This must be confounding the matter and I wonder if the modest difference in good glycaemic control could be partially attributable to this.

I am also concerned on the recommendations for tighter HbA1c control. The features of frailty and functionality are more important in octogenerians and I would not want individuals to interpret this as everyone needs tighter control risking hypos.

**********

6. PLOS authors have the option to publish the peer review history of their article (what does this mean?). If published, this will include your full peer review and any attached files.

If you choose “no”, your identity will remain anonymous but your review may still be made public.

**Do you want your identity to be public for this peer review?**  For information about this choice, including consent withdrawal, please see our Privacy Policy.

Reviewer #1: No

Reviewer #2: **Yes:** Sanjana Gupta

**********

[NOTE: If reviewer comments were submitted as an attachment file, they will be attached to this email and accessible via the submission site. Please log into your account, locate the manuscript record, and check for the action link "View Attachments". If this link does not appear, there are no attachment files.]

To ensure your figures meet our technical requirements, please review our figure guidelines: https://journals.plos.org/plosone/s/figures

You may also use PLOS’s free figure tool, NAAS, to help you prepare publication quality figures: https://journals.plos.org/plosone/s/figures#loc-tools-for-figure-preparation.

NAAS will assess whether your figures meet our technical requirements by comparing each figure against our figure specifications.

# Author comment: Author response to Decision Letter 1

**DOI:** 10.1371/journal.pone.0353388.r002

13 May 2026

We thank the Editor for the opportunity to revise the manuscript. We respond below point by point to the reviewers’ comments.

Reviewer #1:

Q: The question is whether only one annual HbA1c measurement can speak about glycemic control in these patients?

ANSWER: We thank the reviewer for this important comment.

We would like to clarify that the requirement of at least one HbA1c measurement per year was established as a minimum inclusion criterion to ensure adequate follow-up, rather than to define glycemic control based on a single measurement. In fact, glycemic control in our study was determined using multiple HbA1c values over the entire follow-up period, allowing for a longitudinal assessment rather than relying on a single time point.

Consistent with routine clinical practice, patients in our cohort had more frequent measurements than the minimum required. Specifically, the mean number of HbA1c determinations was 1.53 per patient-year, resulting in multiple measurements per individual over the 5-year follow-up period. This approach strengthens the reliability of our classification of glycemic control and reduces the risk of misclassification due to isolated values.

To improve clarity, we have revised the Methods section to better explain how HbA1c measurements were used to define glycemic control over time.

Please see modifications in the first paragraph of the Objectives and Variables section.

Q: Immediately upon reading the instructions, the fear of possible biases (the influence of other factors on glycemia and kidney function, especially in this fragile population) arises. Searching the literature, I find that similar topics have already been covered. It is clear that generally poor glucoregulation implies a less functioning kidney. But in the elderly, it can be due to a number of other reasons besides the glycemic profile.

ANSWER: We fully agree that in older populations, particularly among frail individuals, both glycemic control and kidney function are influenced by multiple factors beyond glucose levels alone.To address this concern, we performed multivariable analyses adjusting for a broad set of clinically relevant covariates, including age, sex, year of inclusion, comorbidities (dyslipidemia, hypertension, obesity, smoking, and alcohol consumption), and pharmacological treatments (statins, ACE inhibitors, angiotensin receptor blockers, oral antidiabetics, NSAIDs, and insulin). We also agree that the association between poor glycemic control and renal impairment has been well established in the general adult population. However, evidence specifically focused on individuals aged ≥80 years remains scarce. In this context, our study contributes by examining this relationship in a very old population using a longitudinal design. Our findings do not suggest that glycemic control is the sole determinant of renal function in older adults, but rather that it remains a relevant factor within a complex clinical framework. This perspective has been further emphasized in the revised Conclusion section, where we explicitly acknowledge the multifactorial nature of CKD development in this population.

Please see changes in the conclusion section.

Q: Keywords: Somehow "diabetes control" and "glycemic control" sound the same, try to be more imaginative.

ANSWER: We thank the reviewer for this suggestion. We have revised the list of keywords to avoid duplication and improve specificity.

Q: Maybe instead of "renal function" in the title, write "development of renal dysfunction or failure or similar"? Just a suggestion.

ANSWER: We thank the reviewer for this suggestion. We agree that the wording of the outcome in the title can be refined to better reflect the clinical nature of the study. In line with this comment, we have revised the title to improve clarity and better represent the focus on CKD development.

Q: The binary division of glycemic control: black/white is also delicate. So, someone with an HbA1c of 7.4 has good control, while someone with 7.5 has bad control. It would be ideal to divide HbA1c differences of, for example, 0.5 into groups or classes in some other way to avoid such exclusivity, so we would get more credible results.

Or as ACCORD maybe take below 7 and above 8 and let's see for those subgroups. Again, just as a suggestion.

ANSEWER: We thank the reviewer for this thoughtful comment regarding the categorization of glycemic control.

We agree that dichotomizing HbA1c values may oversimplify a continuous biological variable and could potentially lead to misclassification, particularly for individuals close to the selected threshold. This is a recognized limitation of categorical approaches and has been acknowledged in the revised Discussion section.

However, our decision to use a binary classification with a threshold of 7.5% was based on clinical and methodological considerations. First, current international guidelines for older adults with type 2 diabetes (including ADA, IDF, and AGS) recommend individualized HbA1c targets within a relatively broad range (approximately 7.0–8.5%), rather than strict universal cut-offs. In this context, 7.5% represents a clinically meaningful and widely accepted intermediate target, particularly for older patients with intermediate health status. Additionally, this threshold is consistent with evidence from major trials such as ACCORD, where mean HbA1c levels around 7.5% were associated with safer outcomes compared to more intensive strategies.

Second, the use of a binary classification facilitates clinical interpretability and allows for a clearer comparison between broadly defined “good” and “poor” glycemic control in a population where treatment goals are often simplified in routine practice.

We acknowledge the reviewer’s suggestion of using multiple categories (e.g., 0.5% intervals or broader ranges such as <7%, 7–8%, >8%), which could provide a more granular understanding of the relationship between HbA1c and CKD risk. However, further stratification would have reduced the sample size within each subgroup and potentially limited statistical power, particularly in this specific population of patients aged ≥80 years.

To address this concern, we have clarified the rationale for the chosen cut-off in the Methods and Discussion sections and explicitly acknowledged the potential limitations of dichotomization. We agree that future studies with larger samples should explore HbA1c as a continuous variable or using multiple clinically relevant categories to better characterize dose–response relationships.

Please see modifications in the Objectives and Variables section, and the third paragraph of the discussion

Q: Is it by chance that the Abstract, Methods section, is written in a different font in the manuscript?

ANSWER: We thank the reviewer for this comment. Any formatting differences in the reviewed version were unintentional. We have carefully revised the manuscript to ensure a consistent format throughout in the resubmitted version.

Q: Please avoid writing abbreviations at the beginning of sentences.

ANSWER: Thank you, we have now avoided this throughout the text.

Q: Nicely written text but full of possible biases. We cannot draw such conclusions based on the analyzed data and without excluding potential other factors.

ANSWER: We thank the reviewer for this important comment.

We agree that, given the observational nature of our study and the potential for residual confounding, the findings should be interpreted with caution and should not be considered as establishing a causal relationship. In response to this comment, we have revised the manuscript to ensure that the conclusions are more appropriately framed in terms of association rather than causation.

We have also strengthened the Discussion section to further acknowledge potential sources of bias and unmeasured confounding factors that may influence the observed relationship.

Please see changes in the Conclusion section of the manuscript and the Conclusion section of the abstract.

Q: It is certainly interesting that there were slightly more smokers and comorbidities in the group with better glucoregulation. Perhaps due to better awareness of the disease? And more ACEi and NSAID users, and better glucoregulation and eGFR. I like that we have that data.

ANSWER: We thank the reviewer for this insightful observation.

We agree that some baseline characteristics, including a slightly higher prevalence of certain comorbidities, smoking, and the use of medications such as ACE inhibitors and NSAIDs in the group with better glycemic control, may appear counterintuitive. One possible explanation could be that patients with a higher burden of comorbidities have more frequent contact with healthcare services, which may facilitate closer monitoring and optimization of glycemic management. However, this interpretation remains speculative.

Importantly, these variables were included in the multivariable analyses, and the association between glycemic control and CKD outcomes remained significant after adjustment. Therefore, while these differences are of interest, they are unlikely to fully explain the observed results.

We have added a sentence in line with the reviewer’s comment in the discussion. Please see changes in the first paragraph of the discussion

Q: P16 L 286: "o" is probably missing an "r" (or).

ANSWER: Corrected, many thanks.

Q: Finally, when I read everything, I congratulate you because this work has value.

ANSWER: We thank the reviewer for this positive and encouraging comment.

We are grateful for the reviewer’s insightful comments, which have contributed to improving the clinical interpretation and balance of our work.

Reviewer #2: Overall a useful question to try to answer.

Q: I don't understand why you chose the cutoff HbA1c 7.5% to differentiate between poor 251 and good glycemic control. It seems like this was selected maybe based to get positive results? Most guidelines suggest <7% if risk of hypos and in T2DM.

ANSWER: While some guidelines suggest targets below 7% for younger or healthier individuals, most international recommendations (including those from the American Diabetes Association, American Geriatrics Society, and International Diabetes Federation) emphasize individualized glycemic targets in older patients, typically within a broader range of approximately 7.0% to 8.5%, depending on comorbidity burden, functional status, and risk of hypoglycemia. In this context, a target around 7.5% is frequently considered appropriate for older adults with intermediate health status and is also consistent with the mean HbA1c achieved in large clinical trials such as ACCORD [21] (N Engl J Med 2008;358:2545–59 ). Specifically, we chose the cutoff point according to the recommendations of the American Diabetes Association, which suggests <7.0-7.5 in otherwise healthy elderly people and <8.0 in those with diabetes and intermediate or complex health [18](Diabetes Care. 2024 Jan 1;47)

In response to the reviewer's comment, we have cited in the Methods section the clinical guideline used to determine the cutoff point.

Please see the first paragraph of the Objectives and Variables section.

Q: Other issues is the strongest confounder in octogenerians will be IHD / heart failure. There is no data on this.

ANSWER: We thank the reviewer for this important observation.

We agree that ischemic heart disease and heart failure are highly prevalent conditions in octogenarians and may act as relevant confounders in the relationship between glycemic control and renal outcomes. These conditions are strongly associated with both kidney function decline and clinical decision-making regarding glycemic targets, and therefore could influence both the exposure and the outcome.

Unfortunately, detailed data on ischemic heart disease and heart failure were not consistently available in the databases used for this study, which limited our ability to include these variables in the multivariable models. However, we attempted to partially account for cardiovascular burden by adjusting for related variables such as hypertension, dyslipidemia, and pharmacological treatments.

We acknowledge that residual confounding due to unmeasured cardiovascular conditions cannot be excluded and may have influenced the observed associations. This limitation has now been explicitly addressed in the Discussion section.

Importantly, despite this limitation, the association between glycemic control and CKD incidence remained significant after adjustment for multiple clinically relevant covariates, suggesting that glycemic control may still play an independent role. Nevertheless, future studies including more detailed cardiovascular data are warranted to further clarify this relationship.

Please see the modifications in the limitations section, sixth paragraph of the discussion.

Q: Another big concern is the absence or acknowledgement of any proteinuria data. This is known to have a strong impact on CKD progression. I understand may be unavailable due to historical data but you at least need to acknowledge this.

ANSWER: We agree that proteinuria is a clinically relevant factor in the progression of chronic kidney disease and would have been informative to describe in our study population. Unfortunately, proteinuria data were not consistently available in the databases used.

From an analytical perspective, the role of proteinuria in this context is complex. While it may be associated with both glycemic control and renal outcomes, it is also likely to lie along the causal pathway linking hyperglycemia and kidney function decline. Therefore, adjusting for proteinuria could potentially attenuate the association of interest.

For this reason, and given the lack of consistent data, proteinuria was not included in the models. We have clarified this point and acknowledged the absence of proteinuria data in the Discussion section.

In line with the reviewer's comment, we have added a paragraph to the discussion. Please see the 7th paragraph of the discussion.

Q: I am also concerned that the good control ACEi / ARB use was 11.32% vs 6.83% in poor control. This is a difference of 4.5% in ACEi /ARB use. This must be confounding the matter and I wonder if the modest difference in good glycaemic control could be partially attributable to this.

ANSWER: We thank the reviewer for this observation.

First, we would like to clarify the figures reported for ACE inhibitors (ACEi) and angiotensin receptor blockers (ARBs) in our study population. According to Table 2, the use of ACE inhibitors was 4.83% in the poor glycemic control group and 8.13% in the good control group, while ARB use was 2.30% and 3.19%, respectively. Therefore, although there is a slightly higher use of these medications in the good control group, the absolute differences are relatively small.

Importantly, both ACE inhibitors and ARBs were included as covariates in the multivariable regression models (both logistic and Cox regression analyses). After adjustment for these treatments, the association between glycemic control and CKD incidence, as well as time to CKD onset, remained statistically significant. This suggests that the observed relationship is unlikely to be fully explained by differences in ACEi/ARB use.

We agree that these medications may have a protective effect on renal outcomes and could act as confounders. For this reason, they were specifically included in the adjusted models. Nevertheless, as with any observational study, residual confounding cannot be entirely excluded and has been acknowledged in the Discussion section.

Please see changes in the 6th paragraph of the discussion, dedicated to limitations, we have mentioned ACE inhibitors and ARBs among the potential confounding factors that we have sought to control.

Q: I am also concerned on the recommendations for tighter HbA1c control. The features of frailty and functionality are more important in octogenerians and I would not want individuals to interpret this as everyone needs tighter control risking hypos.

ANSWER: We fully agree with the reviewer and believe that a study with the characteristics of ours should not be interpreted in isolation as a basis

# Editor report: Decision Letter 1

**DOI:** 10.1371/journal.pone.0353388.r003

**Authors:** Natural Hoi Sing Chu, Natural Hoi Sing Chu

24 Jun 2026

Association Between Glycemic Control and Chronic Kidney Disease Development in Older Patients with Type 2 Diabetes: A Retrospective Cohort Study

PONE-D-25-64900R1

Dear Dr. Rodríguez-Molinero,

We’re pleased to inform you that your manuscript has been judged scientifically suitable for publication and will be formally accepted for publication once it meets all outstanding technical requirements.

Within one week, you’ll receive an e-mail detailing the required amendments. When these have been addressed, you’ll receive a formal acceptance letter and your manuscript will be scheduled for publication.

An invoice will be generated when your article is formally accepted. Please note, if your institution has a publishing partnership with PLOS and your article meets the relevant criteria, all or part of your publication costs will be covered. Please make sure your user information is up-to-date by logging into Editorial Manager at Editorial Manager® and clicking the ‘Update My Information' link at the top of the page. For questions related to billing, please contact billing support.

If your institution or institutions have a press office, please notify them about your upcoming paper to help maximize its impact. If they’ll be preparing press materials, please inform our press team as soon as possible -- no later than 48 hours after receiving the formal acceptance. Your manuscript will remain under strict press embargo until 2 pm Eastern Time on the date of publication. For more information, please contact onepress@plos.org.

Kind regards,

Natural Hoi Sing Chu, Ph.D

Academic Editor

PLOS One

Additional Editor Comments (optional):

Reviewers' comments:

# Editor report: Acceptance letter

**DOI:** 10.1371/journal.pone.0353388.r004

**Authors:** Natural Hoi Sing Chu, Natural Hoi Sing Chu

PONE-D-25-64900R1

PLOS One

Dear Dr. Rodríguez-Molinero,

I'm pleased to inform you that your manuscript has been deemed suitable for publication in PLOS One. Congratulations! Your manuscript is now being handed over to our production team.

At this stage, our production department will prepare your paper for publication. This includes ensuring the following:

* All references, tables, and figures are properly cited

* All relevant supporting information is included in the manuscript submission,

* There are no issues that prevent the paper from being properly typeset

You will receive further instructions from the production team, including instructions on how to review your proof when it is ready. Please keep in mind that we are working through a large volume of accepted articles, so please give us a few days to review your paper and let you know the next and final steps.

Lastly, if your institution or institutions have a press office, please let them know about your upcoming paper now to help maximize its impact. If they'll be preparing press materials, please inform our press team within the next 48 hours. Your manuscript will remain under strict press embargo until 2 pm Eastern Time on the date of publication. For more information, please contact onepress@plos.org.

You will receive an invoice from PLOS for your publication fee after your manuscript has reached the completed accept phase. If you receive an email requesting payment before acceptance or for any other service, this may be a phishing scheme. Learn how to identify phishing emails and protect your accounts at https://explore.plos.org/phishing.

If we can help with anything else, please email us at customercare@plos.org.

Thank you for submitting your work to PLOS ONE and supporting open access.

Kind regards,

PLOS ONE Editorial Office Staff

on behalf of

Dr. Natural Hoi Sing Chu

Academic Editor

PLOS One
